# Supplementary material for: Lipid nanoparticles with PEG-variant surface modifications mediate genome editing in the mouse retina
Source: Nat Commun. 2023 Oct 13;14:6468. doi: 10.1038/s41467-023-42189-3 (PMC10575971; doi:10.1038/s41467-023-42189-3)
Supplement: Supplementary file 1 — Supplementary file [file 41467_2023_42189_MOESM1_ESM.pdf]

Supplementary Information

Lipid nanoparticles with PEG-variant surface modifications mediate genome editing in the mouse retina

Milan Gautam[a], Antony Jozic[a], Grace Li-Na Su[b], Marco Herrera-Barrera[a], Allison Curtis[b], Sebastian Arrizabalaga[b], Wayne Tschetter[b], Renee C. Ryals[b*], Gaurav Sahay[a,b,c*]

[a]Department of Pharmaceutical Sciences, College of Pharmacy, Robertson Life Sciences Building, Oregon State University, Portland, Oregon, 97201, USA

[b]Department of Ophthalmology, Casey Eye Institute, Oregon Health & Science University, Portland, Oregon, 97239, USA

[c]Department of Biomedical Engineering, Robertson Life Sciences Building, Oregon Health & Science University, Portland, Oregon, 97201, USA

*Correspondence to: Gaurav Sahay; email: sahay@ohsu.edu
Renee C. Ryals; email: ryals@ohsu.edu

This file includes:

Supplementary Figures 1-7

Supplementary Tables 1-4

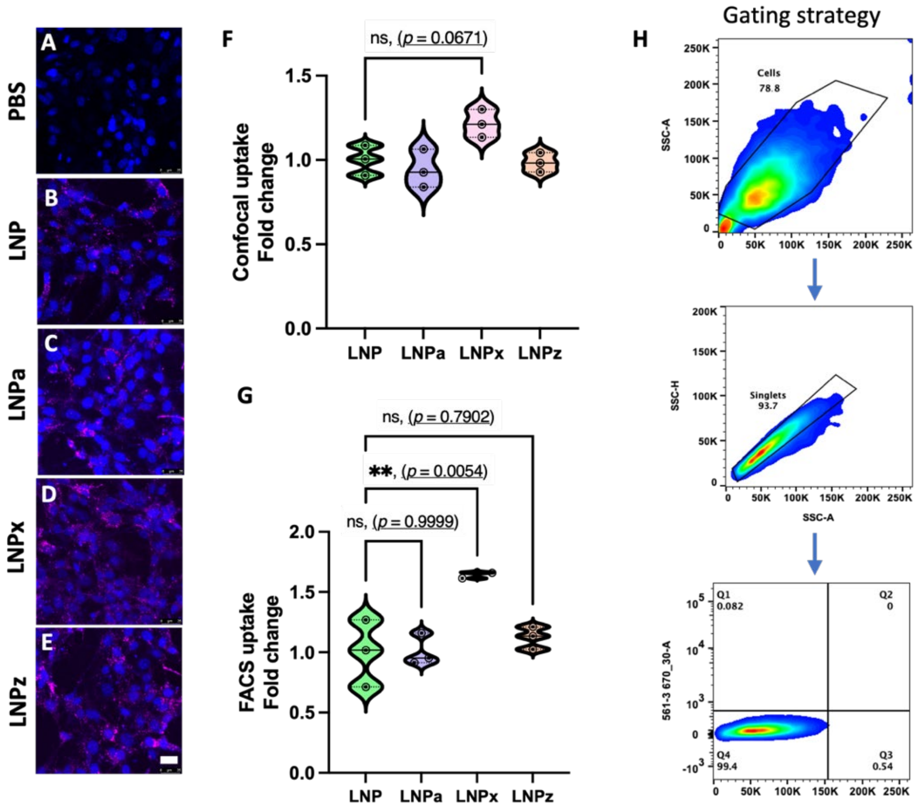

Supplementary Figure 1. Intracellular uptake of Cy-5 tagged LNP variants. (A-E) Representative confocal images of 661W cone cells treated with Cy-5 tagged LNP variants at 24 hours treatment. All the cells were treated with Cy5-tagged LNP dose equivalent to 100 ng/well of mRNA cargo (scale bar: 25 µm). **(F)** Relative cellular uptake of Cy-5 tagged LNP variants obtained by quantitative estimation of confocal images against unmodified LNP. **(G)** Quantification of cellular uptake using flow cytometry. **(H)** Gating strategy used during cellular uptake study for untreated cells and same strategy was used for other LNP variant treated cells. All data are presented as Mean ± SD. An ordinary one-way ANOVA, with Tukey's correction for multiple comparisons test was used for comparisons. Unmodified LNP values were normalized, and fold changes were compared with LNP variants. ns-not significant, $^{**}p<0.01$. All representative figures and graphs have n=3 replicates. Source data are provided as a Source Data file.

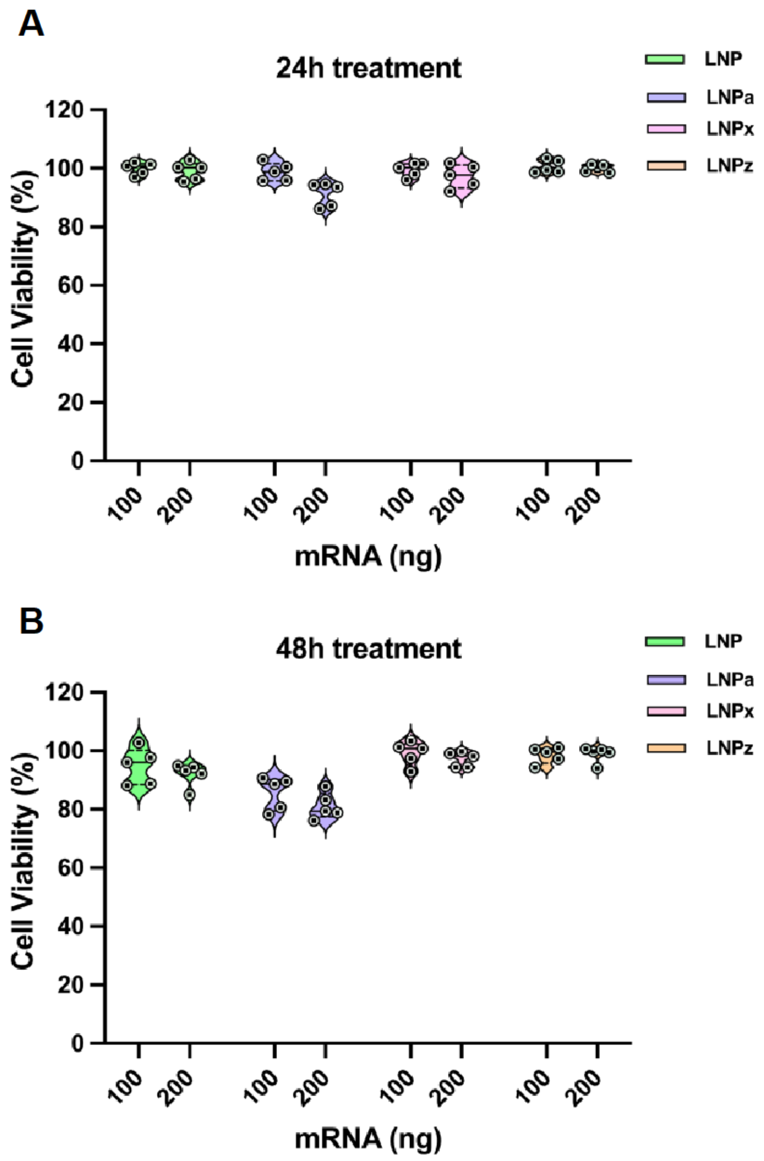

Supplementary Figure 2. ***In-vitro*** **cell viability assay.** 661w cone cells were treated with LNP variants at a 100 and 200 ng fluc mRNA dose. **(A)** 24 hours post-treatment and **(B)** 48 hours post-treatment. All graphs have n=5 replicates. Source data are provided as a Source Data file.

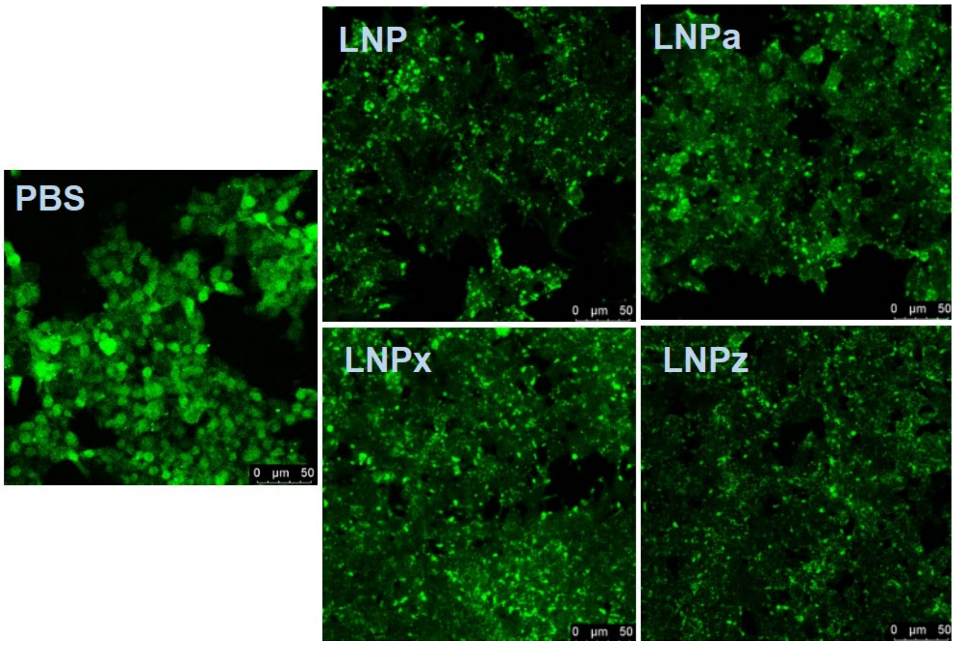

Supplementary Figure 3. Endosomal escape of LNP variants. Representative images of the Gal8-GFP reporter cells, 24 hours after *cre* mRNA-loaded LNP variant treatments. All representative figures from n = 3 replicates.

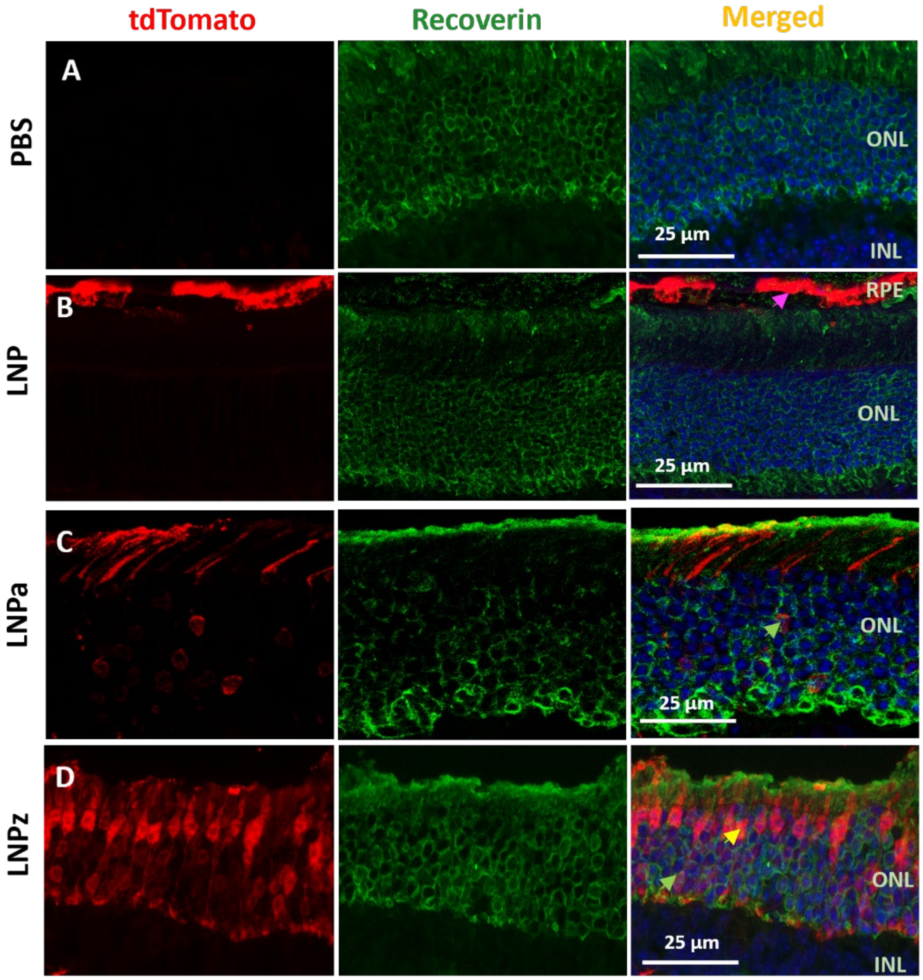

Supplementary Figure 4. Photoreceptor colocalization study in Ai9 mice. (A-D) Representative confocal images showing tdTomato expression in the RPE (LNP) and photoreceptors (LNPa and LNPz), which are colocalized with recoverin. Arrow indicates tdTomato expression (pink: RPE, yellow: cone photoreceptor nuclei, green: rod photoreceptor nuclei). RPE: retinal pigment epithelium, ONL: outer nuclear layer, INL: inner nuclear layer. All representative figures from n = 6 single eyes per group.

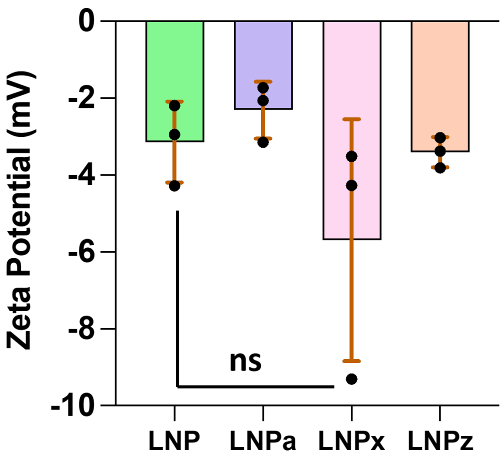

Supplementary Figure 5. Zeta potential value from mCherry encapsulated LNP variants measured by Malvern zeta sizer. n=3 LNPs per group. All Data are presented as Mean ± SD. An ordinary one-way ANOVA, with Tukey's correction was used for comparison with unmodified LNP. ns-not significant. Source data are provided as a Source Data file.

A) A*A*G*UAAAACCUCUACAAAUGGUUUUAGAGCUAGAAAUAGCAAGUUAA
AAUAAGGCUAGUCCGUUAUCAACUUGAAAAAGUGGCACCGAGUCGGUG
CU*U*U*U

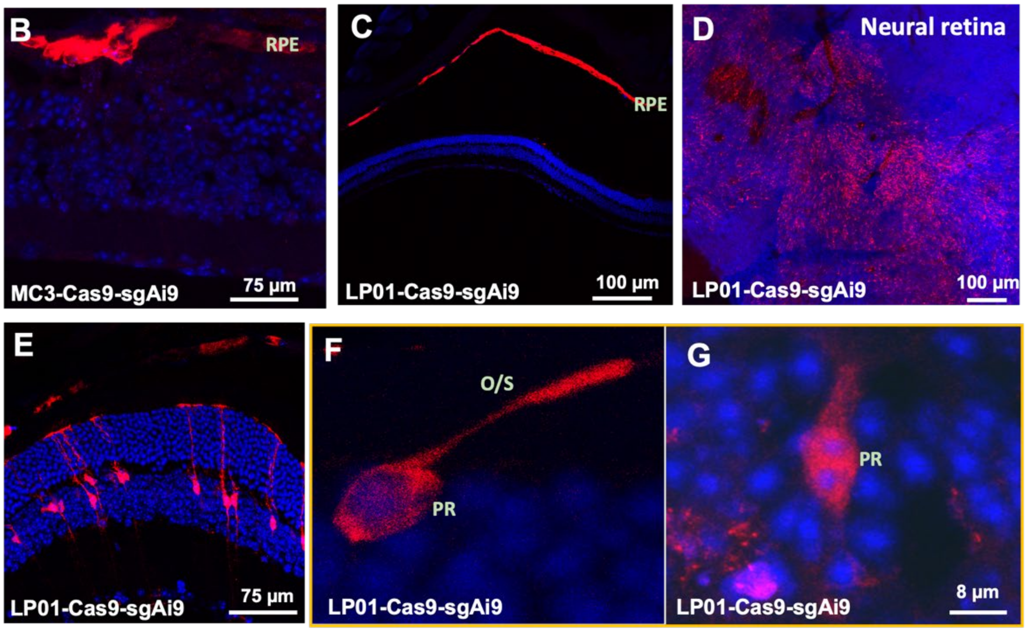

Supplementary Figure 6. CRISPR-Cas9 delivery and sgRNA modification in Ai9 mice. (A) sgAi9 guide with modifications highlighted. In the sgRNA structure, the asterisks (blue) and red font indicate the phosphorothioate bond and 2′-O-methyl ribonucleotides, respectively. **(B)** Representative confocal image showing outer nuclear thinning and disruption 7-days post-subretinal injection with MC3-Cas9-sgAi9-LNPx (n=6 single eyes). **(C-G)** Representative confocal images showing editing events post-LP01-Cas9-sgAi9-LNPx injection. **(C)** Retinal cross section highlighting RPE editing (n=6 single eyes). **(D)** Neural retina flatmount showing tdTomato fluorescence signal within the treated area of the retina (n=9 single eyes). **(E)** Retinal cross sections highlighting editing in the Müller glia and **(F-G)** photoreceptors (n=6 single eyes). O/S: outer segment, PR: photoreceptor, RPE: retinal pigment epithelium.

A

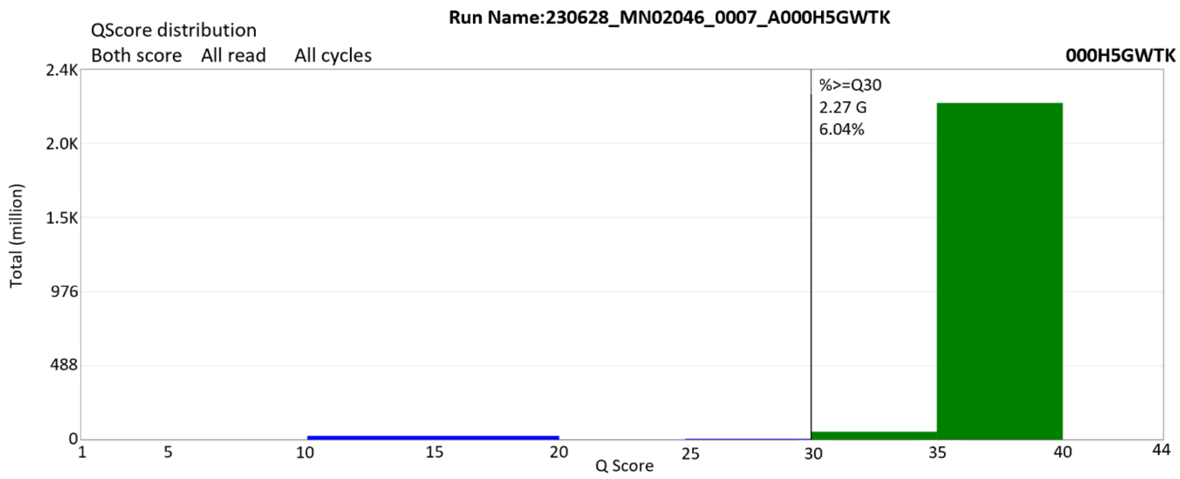

B

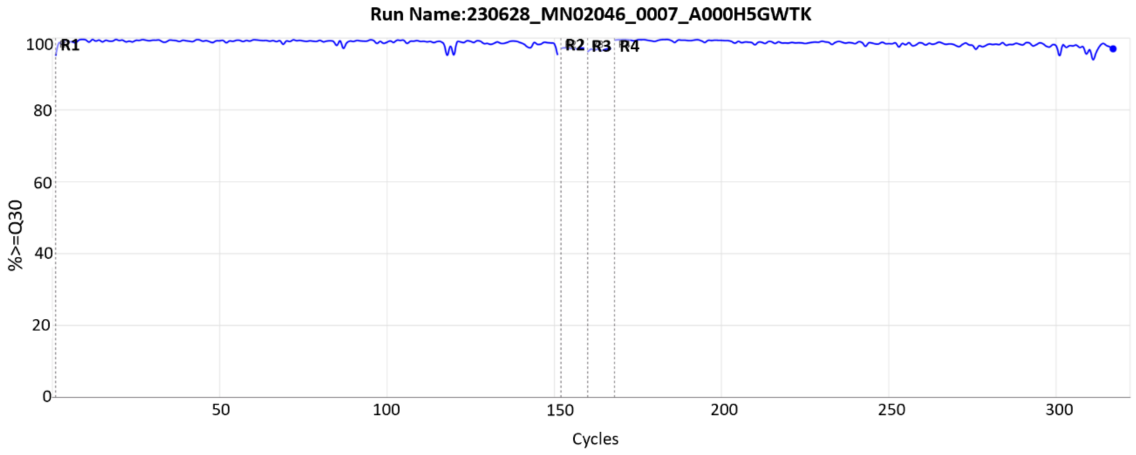

Supplementary Figure 7. Illumina quality control data from NGS. (A) QScore distribution indicating high quality sequencing data (over 96% Q30 and above). **(B)** Percentage of bases with >= Q30 plotted by cycle showing high accuracy of base call across the whole sequencing run (n=9).

Supplementary Table 1: Antibodies used for staining

S.N.	Primary Antibody	Host	Dilution
1.	Anti-recoverin (Cat. # AB5585)	Anti-rabbit	1:500
2.	Anti-mCherry (Cat. # NBP2-25157)	Anti-rabbit	1:250
3.	Visual Arrestin (E-3) (SC-166388)	Anti-mouse	1:100

S.N.	Secondary Antibody	Host	Dilution
1.	Alexa Fluor 700-IgG (Cat. # A21038)	Goat anti-rabbit	1:1000
2.	Alexa Fluor 594-IgG (H+L) (Cat. # A-21207)	Donkey anti-rabbit	1:500
3.	Alexa Fluor Plus 488-IgG (H+L) (Cat. # A21038)	Goat anti-mouse	1:200

Supplementary Table 2: Guide RNA, primers, and cycling conditions used for editing studies

S.N.	sgRNA Name	Sequence
1.	sgAi9	AAGUAAAACCUCUACAAAUG
2.	sgGFP	CUCGUGACCACCCUGACCUA

S.N.	Primer Name	Sequence (5' -> 3')
1.	Ai9_NGS_F1_F	ACACTCTTTCCCTACACGACGCTCTTCCGATCTATACGAAGTTATTCGCGATG
2.	Ai9_NGS_F2_F	ACACTCTTTCCCTACACGACGCTCTTCCGATCTGTTGTGGTTTGTCCAAAC
3.	Ai9_NGS_R	GACTGGAGTTCAGACGTGTGCTCTTCCGATCTTGTTTCAGGTTCAGGGGGAG

Supplementary Table 3: Thermocycling methods for 1st PCR for NGS library preparation

Step	Temperature	Time
Initial Denaturation	95.0 °C	3 min
30 Cycles:<br>Denaturation<br>Annealing<br>Extension	 98.0 °C<br>60.0 °C<br>72.0 °C	 20 sec<br>20 sec<br>30 sec
Final Extension	72.0 °C	30 sec

Supplementary Table 4: Thermocycling methods for 2nd PCR for NGS library preparation

Step	Temperature	Time
Initial Denaturation	98.0 °C	2 min
12 Cycles:<br>Denaturation<br>Annealing<br>Extension	 98.0 °C<br>62.0 °C<br>72.0 °C	 10 sec<br>20 sec<br>30 sec
Final Extension	72.0 °C	2 min
