# Supplementary material for: Lipid nanoparticles with PEG-variant surface modifications mediate genome editing in the mouse retina
Source: Nat Commun. 2023 Oct 13;14:6468. doi: 10.1038/s41467-023-42189-3 (PMC10575971; doi:10.1038/s41467-023-42189-3)
Supplement: Supplementary file 3 — Reporting Summary [file 41467_2023_42189_MOESM3_ESM.pdf]

nature portfolio

Corresponding author(s): Gaurav Sahay & Renee C. Ryals

Last updated by author(s): Sep 27, 2023

Reporting Summary

Nature Portfolio wishes to improve the reproducibility of the work that we publish. This form provides structure for consistency and transparency in reporting. For further information on Nature Portfolio policies, see our Editorial Policies and the Editorial Policy Checklist.

Statistics

For all statistical analyses, confirm that the following items are present in the figure legend, table legend, main text, or Methods section.

n/a	Confirmed	
☐	☒	The exact sample size (*n*) for each experimental group/condition, given as a discrete number and unit of measurement
☐	☒	A statement on whether measurements were taken from distinct samples or whether the same sample was measured repeatedly
☐	☒	The statistical test(s) used AND whether they are one- or two-sided<br>*Only common tests should be described solely by name; describe more complex techniques in the Methods section.*
☒	☐	A description of all covariates tested
☒	☐	A description of any assumptions or corrections, such as tests of normality and adjustment for multiple comparisons
☐	☒	A full description of the statistical parameters including central tendency (e.g. means) or other basic estimates (e.g. regression coefficient) AND variation (e.g. standard deviation) or associated estimates of uncertainty (e.g. confidence intervals)
☐	☒	For null hypothesis testing, the test statistic (e.g. *F*, *t*, *r*) with confidence intervals, effect sizes, degrees of freedom and *P* value noted<br>*Give P values as exact values whenever suitable.*
☒	☐	For Bayesian analysis, information on the choice of priors and Markov chain Monte Carlo settings
☒	☐	For hierarchical and complex designs, identification of the appropriate level for tests and full reporting of outcomes
☒	☐	Estimates of effect sizes (e.g. Cohen's *d*, Pearson's *r*), indicating how they were calculated

Our web collection on statistics for biologists contains articles on many of the points above.

Software and code

Policy information about availability of computer code

Data collection	Leica DMi8, BD LSRFortessaTM Cell Analyzer, Illumina MiniSeq
Data analysis	Fiji ImageJ2 version: 2.9.0/1.53t, GraphPad Prism version: 9.5.0, FlowJo 10.8.1, CRISPResso2 (v2.2.14)

For manuscripts utilizing custom algorithms or software that are central to the research but not yet described in published literature, software must be made available to editors and reviewers. We strongly encourage code deposition in a community repository (e.g. GitHub). See the Nature Portfolio guidelines for submitting code & software for further information.

Data

Policy information about availability of data

All manuscripts must include a data availability statement. This statement should provide the following information, where applicable:

- Accession codes, unique identifiers, or web links for publicly available datasets
- A description of any restrictions on data availability
- For clinical datasets or third party data, please ensure that the statement adheres to our policy

The authors declare that all other data supporting the findings of this study are available within the paper and its Supplementary Information files. Sequencing data is available from the Sequence Read Archive under accession code PRJNA991562 (https://www.ncbi.nlm.nih.gov/bioproject/PRJNA991562). Source data are provided with this paper.

Research involving human participants, their data, or biological material

Policy information about studies with human participants or human data. See also policy information about sex, gender (identity/presentation), and sexual orientation and race, ethnicity and racism.

Reporting on sex and gender	N/A
Reporting on race, ethnicity, or other socially relevant groupings	N/A
Population characteristics	N/A
Recruitment	N/A
Ethics oversight	N/A

Note that full information on the approval of the study protocol must also be provided in the manuscript.

Field-specific reporting

Please select the one below that is the best fit for your research. If you are not sure, read the appropriate sections before making your selection.

☒ Life sciences ☐ Behavioural & social sciences ☐ Ecological, evolutionary & environmental sciences

For a reference copy of the document with all sections, see nature.com/documents/nr-reporting-summary-flat.pdf

Life sciences study design

All studies must disclose on these points even when the disclosure is negative.

Sample size	Sample sizes were determined based on literature precedent, ethical considerations (using the minimum number of animals needed for experimentation), and allowable error size, accuracy, resources, and the need for statistical analysis (at least n = 3 throughout all the studies). No statistical methods were used to determine the sample size in the study. The sample size is clearly described in each figure legend. The number of animals is the minimum that we require, based on historical data, to generate reliable data while maintaining our commitment to the 3 R's.
Data exclusions	No animal and/or data were excluded.
Replication	Three or more independent studies were carried out. All the attempts at replication were successful, and the standard deviation was within the expected range.
Randomization	For in vitro studies, all the samples were randomly allocated into experimental groups, as there was no covariate in the study design. Each mouse was allocated to each experimental condition by investigators. Each eye received an equal dose of mRNA; thus, no randomization was used during the in vivo study.
Blinding	The investigators were not blinded during animal experiments, data collection, or analysis. Sub-retinal needs highly skilled personnel for injection, and all of the procedures, like topical administration of drugs for eye dilation, putting under general anesthesia, and sub-retinal injection, were performed by the same investigator. Blinding the investigator would have been difficult due to the potential injection error risk.

Reporting for specific materials, systems and methods

We require information from authors about some types of materials, experimental systems and methods used in many studies. Here, indicate whether each material, system or method listed is relevant to your study. If you are not sure if a list item applies to your research, read the appropriate section before selecting a response.

Materials & experimental systems

n/a	Involved in the study
	☒ Antibodies
	☒ Eukaryotic cell lines
☒	☐ Palaeontology and archaeology
	☒ Animals and other organisms
☒	☐ Clinical data
☒	☐ Dual use research of concern
☒	☐ Plants

Methods

n/a	Involved in the study
☒	☐ ChIP-seq
	☒ Flow cytometry
☒	☐ MRI-based neuroimaging

Antibodies

Antibodies used	Primary antibodies used were Anti-recoverin (MilliporeSigma, Cat. # AB5585), Anti-mCherry (Novus Biologicals, Cat. # NBP2-25157) and Visual Arrestin E-3 (Santa Cruz, SC-166383). Secondary antibodies used were Alexa Fluor 700-IgG (Invitrogen, Cat. # A21038), Alexa Fluor 594-IgG (H+L) (Invitrogen, Cat. # A-21207) and Alexa Fluor Plus 488-IgG (H+L) (Invitrogen, Cat. # A21038)
Validation	The antibodies have been previously validated by the authors. Anti-recoverin by Han, I.C. et. al. Gene Ther 30, 362–368 (2023), Anti-mCherry by S. Patel et. al. J Control Release 303, 91-100 (2019), Visual Arrestin E-3 by Herrera-Barrera, M. et al. Sci Adv. 9: eadd4623 (2023). The vendor also provides the validation of both primary and secondary antibodies used in this study.

Eukaryotic cell lines

Policy information about cell lines and Sex and Gender in Research

Cell line source(s)	661W cone cells were generously provided by Prof. Muayyad Al-Ubaidi, University of Houston, Houston, TX. Gal8-GFP reporter cells using Human Embryonic Kidney 293T/17 cells (CRL-11268; ATCC, Manassas, VA).
Authentication	661W cone cells were authenticated by seeing cell morphology. GFP-positive Gal8-GFP-HEK 293T/17 cells were already authenticated by Herrera-Barrera, M. et al., Biomater. Sci., 9, 4289 (2021) by using BD FACSAria™ Fusion equipped with a 488nm laser, and we confirmed this by checking morphology and using the GFP channel in confocal microscopy.
Mycoplasma contamination	No mycoplasma contamination was confirmed by the authors.
Commonly misidentified lines (See ICLAC register)	The cell lines used in this work are not in the list of misidentified lines.

Animals and other research organisms

Policy information about studies involving animals; ARRIVE guidelines recommended for reporting animal research, and Sex and Gender in Research

Laboratory animals	Breeder Ai9 (Strain # 007909) mice were purchased from The Jackson Laboratory (Bar Harbor, ME, USA). Breeder NRL-GFP mice were generously endued by Dr. Anand Swaroop. All mice were housed in a specific-pathogen-free animal facility at ambient temperature (22±2°C), air humidity 40–70% and 12-h dark/12-h light cycle. All mice were maintained on a free access to standard rodent chow diet (5L0D - PicoLab) and water. All the mice used in the experiments were 1 to 6 months old and specific age has mentioned in figure label, if applicable. Mice were bred in-house for the experiments.
Wild animals	No wild animals were used in the study.
Reporting on sex	Both male and female mice were used. Male and female mice were pooled and tested collectively throughout the study. The study did not involve separate analyses for each gender, and there was an unequal distribution of male and female mice. No animals or data points were excluded during the experiment or data analysis.
Field-collected samples	No field-collected samples were used in the study.
Ethics oversight	All experimental procedures were carried out in accordance with the protocols approved by Oregon Health & Science University's Institutional Animal Care and Use Committee and in accordance with the Association for Research in Vision and Ophthalmology's (ARVO) Statement.

Note that full information on the approval of the study protocol must also be provided in the manuscript.

Flow Cytometry

Plots

Confirm that:

☒ The axis labels state the marker and fluorochrome used (e.g. CD4-FITC).

☒ The axis scales are clearly visible. Include numbers along axes only for bottom left plot of group (a 'group' is an analysis of identical markers).

☒ All plots are contour plots with outliers or pseudocolor plots.

☒ A numerical value for number of cells or percentage (with statistics) is provided.

Methodology

Sample preparation	661w cones cells were used to check cellular uptake of Cy-5 tagged LNPs using Flow cytometry.
Instrument	BD LSRFortessaTM Cell Analyzer
Software	All data were analyzed by FlowJo 10.8.1 software
Cell population abundance	No purification done.
Gating strategy	Singlets cells were gated and Cy-5 positive cells were analyzed.

☒ Tick this box to confirm that a figure exemplifying the gating strategy is provided in the Supplementary Information.
